# Spontaneous Resolution of Symptomatic Synovial Cysts of the Lumbar Spine: A Comprehensive Review with Two Illustrative Cases

**DOI:** 10.3390/medicina60071115

**Published:** 2024-07-09

**Authors:** Raffaele Scrofani, Matteo De Simone, Filippo Migliorini, Ettore Amoroso, Nicola Maffulli, Nicola Narciso, Giorgio Iaconetta

**Affiliations:** 1 Department of Clinical Neurosurgery, AOU San Giovanni di Dio e Ruggi D′Aragona, University of Salerno, Via San Leonardo 1, 84131 Salerno, Italy; raffaele7@gmail.com (R.S.); ettore.amoroso@sangiovannieruggi.it (E.A.); nicola.narciso@sangiovannieruggi.it (N.N.); 2 Department of Medicine, Surgery and Dentistry “Scuola Medica Salernitana”, University of Salerno, Via S. Allende, 84081 Baronissi, Italy; 3 BrainLab s.r.l., Mercato San Severino, 84085 Salerno, Italy; 4 Department of Orthopaedic, Trauma, and Reconstructive Surgery, RWTH University Hospital, 52074 Aachen, Germany; migliorini.md@gmail.com; 5 Department of Orthopaedics, Faculty of Medicine and Psychology, University of Rome “La Sapienza”, 00185 Rome, Italy; nicola.maffulli@uniroma1.it; 6 School of Pharmacy and Bioengineering, Keele University School of Medicine, Thornburrow Drive, Stoke on Trent ST5 5BG, UK; 7 Barts and the London School of Medicine and Dentistry, Centre for Sports and Exercise Medicine, Queen Mary University of London, Mile End Hospital, 275 Bancroft Road, London E1 4DG, UK

**Keywords:** synovial cysts, spine surgery, spontaneous resolution, lumbar spine, low back pain

## Abstract

Although lumbar synovial cysts (LSCs) are frequently described in the literature, they are a relatively uncommon cause of low back and radicular leg pain. Furthermore, their spontaneous resolution is an even rarer event. The standard treatment of the lumbar synovial cyst is surgical excision. Spontaneous resolution in the literature is a sporadic event. In our experience, we have had two cases where the lumbar synovial cyst disappeared spontaneously. To date, only nine cases of spontaneous resolution of synovial cysts have been documented in the literature. In this discussion, we highlight a pathology that typically suggests surgical intervention, yet conservative treatment can be a viable alternative. We present two cases of large synovial cysts that were initially scheduled for surgery but ultimately resolved spontaneously without any treatment. While the spontaneous resolution of lumbar synovial cysts is extremely rare, conservative strategies are an option that should not be overlooked. Our cases contribute to the growing body of evidence on the spontaneous regression of symptomatic LSC, potentially enhancing the understanding of the disease’s natural progression in the future.

## 1. Introduction

Lumbar spine synovial cysts are benign growths adjacent to the facet joints that can cause lumbar pain, lumbar radiculopathy, and neurological deficits. The first description of this entity goes back to Baker who first described its formation in 1885; however, he had studied them on limb joints, and in particular, his work referred to the knee joint [1]. In 1974, Kao et al. were the first authors to report symptomatic nerve compression secondary to a lumbar synovial cyst and called these juxta facet cysts [2]. Other types include the ganglion cyst (cyst loses continuity with the capsule of the facet joints), ligamentum flavum cyst, and posterior longitudinal ligament cyst.

Diagnostic framing in this context is never easy and obvious, especially if we can intuit how this entity may be superimposed on degenerative disc pathology. The spread of the diagnosis of this nosological entity actually began in the middle of the twentieth century and became established in the late twentieth century when the spread of tomographic diagnostic methods allowed accurate morphological characterization of the disease process.

The pathophysiology is unknown, but one of the most reliable hypotheses found in the literature is that the increased mobility and repetitive microtraumas may produce the extrusion of synovial fluid by the facet joint and the progressive growth of residual myxoid degeneration [3]. The majority of LSC patients are in their sixties and generally present with lumbar degenerative spondylosis, but lumbar synovial cysts are also present in young patients, and trauma is often the cause [3]. Lumbar synovial cysts are known to cause various symptoms and signs, such as painful radiculopathy, neurogenic claudication, and cauda equina syndrome [4]. Spontaneous bleeding into the cyst may also occur, causing an acute deterioration of the symptoms; even an acute cauda syndrome has been described [5]. Synovial cysts typically extend posterolaterally to the thecal sac, often requiring hemilaminectomy or interlaminotomy for complete excision. Some cases of synovial cysts are accompanied by spondylolisthesis, necessitating stabilization to address the underlying instability. Segmental instability of the facet joints has been suggested as an important factor in the etiology of synovial cysts [6]. Although the diagnosis can stem from clinical suspicion, undoubtedly the diagnostic method that is considered the gold standard is MRI with contrast medium, in which the cyst wall takes contrast. The parallel, however, CT scan can also be useful as it provides an opportunity to detect recent bleeding and in parallel can detect facet joint tilt, which is a radiological finding that correlates with instability.

Conservative management includes bed rest, analgesia, bracing, CT-guided percutaneous cyst aspiration, and facet joint and epidural steroid injections. The management of symptomatic LSC is controversial due to the unknown natural history of the disease. While surgery is the most frequent therapeutic option, conservative treatments are also viable. Our clinical cases demonstrate that spontaneous resolution, although considered rare, may be more common than previously believed. Therefore, alongside surgical approaches, conservative management should be considered.

## 2. Case Presentation

**Figure 1:**
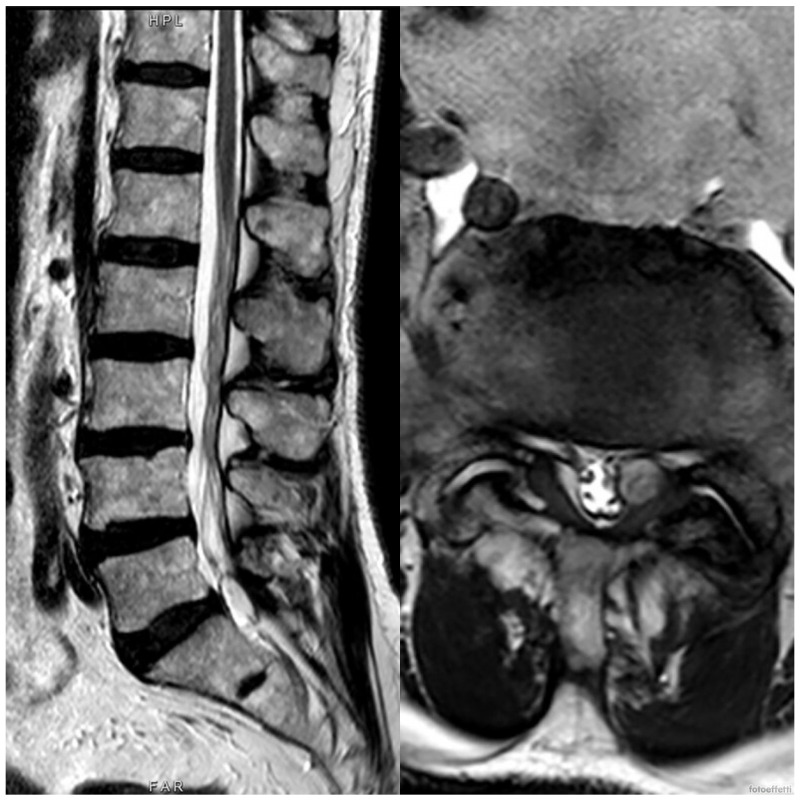
Preoperative MRI of the lumbar spine of the first case, a 66-year-old man, where there is a synovial cyst at L5-S1 level.

Case 1 involved a 66-year-old man who underwent dynamic flexion/extension radiographs to assess the degree of spondylolisthesis along with magnetic resonance imaging (MRI, Figure 1) and computed tomography (CT) with or without myelography of the lumbosacral spine. Radiologic imaging facilitated the final diagnosis, preoperative planning, and the characterization and marking of the level and number of synovial cysts. The patient presented to our institution with a 12-month history of severe back pain and left leg pain in the L5/S1 distribution. The pain was so intense that it interrupted his daily activities with a visual analog scale (VAS) score of 8/10. He reported no other medical problems or previous surgeries. Over time, the pain progressively worsened in both frequency and intensity. Medical therapy failed to alleviate the pain, which had become unbearable.

**Figure 2:**
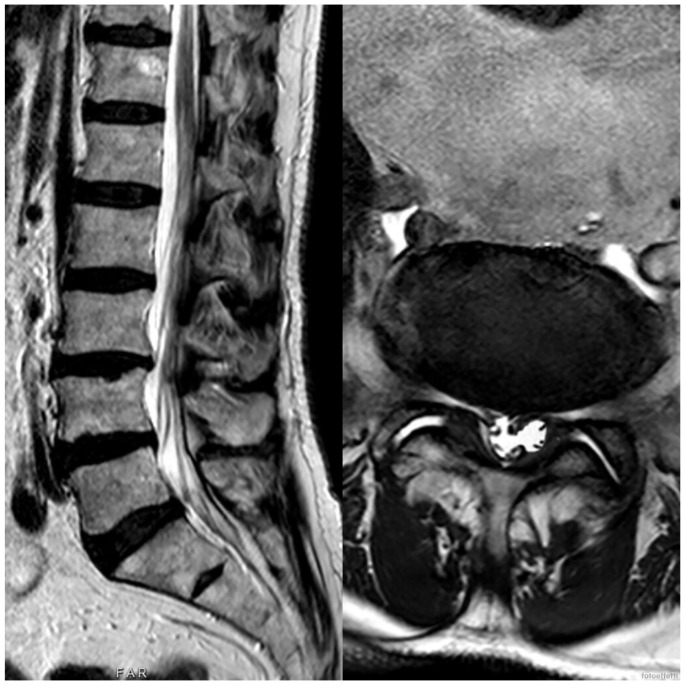
Preoperative MRI of the lumbo-sacral spine of the second patient; here, we can observe the left L4/L5 synovial cyst.

Case 2 was 46-year-old woman. This patient had dynamic flexion/extension radiographs (to assess the degree of spondylolisthesis) and magnetic resonance imaging (Figure 2) of the lumbosacral spine. The 46-year-old woman presented to our attention with left lumbosciatica in l4–l5. Pharmacological therapy was useless. The pain was unbearable with a VAS score of 9/10.

**Figure 3:**
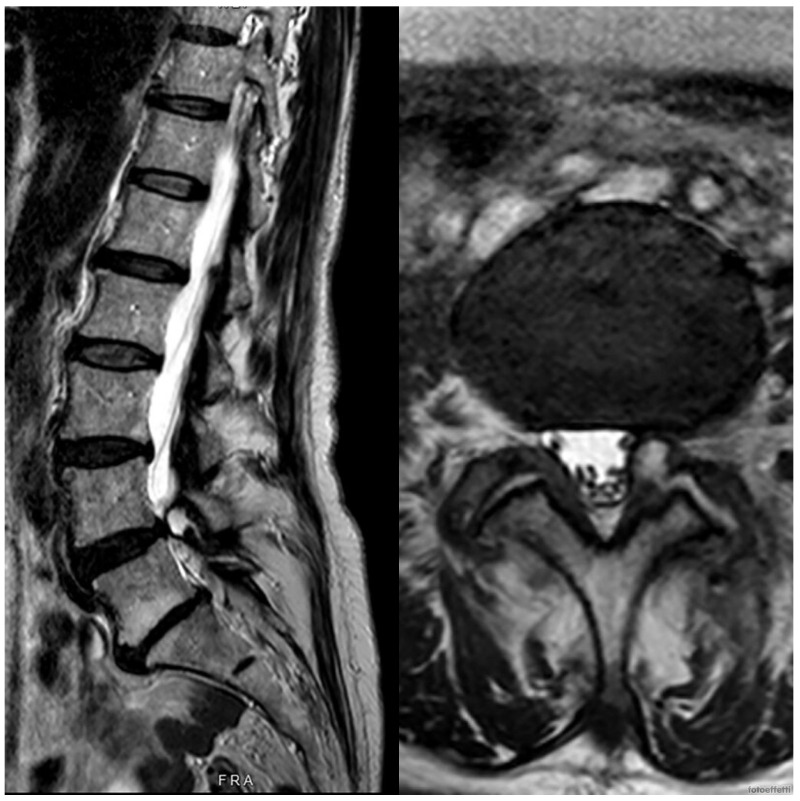
In this MRI of the lumbar spine of the first case after about 3 months, we can see the spontaneous reabsorption of the synovial cyst.

**Figure 4:**
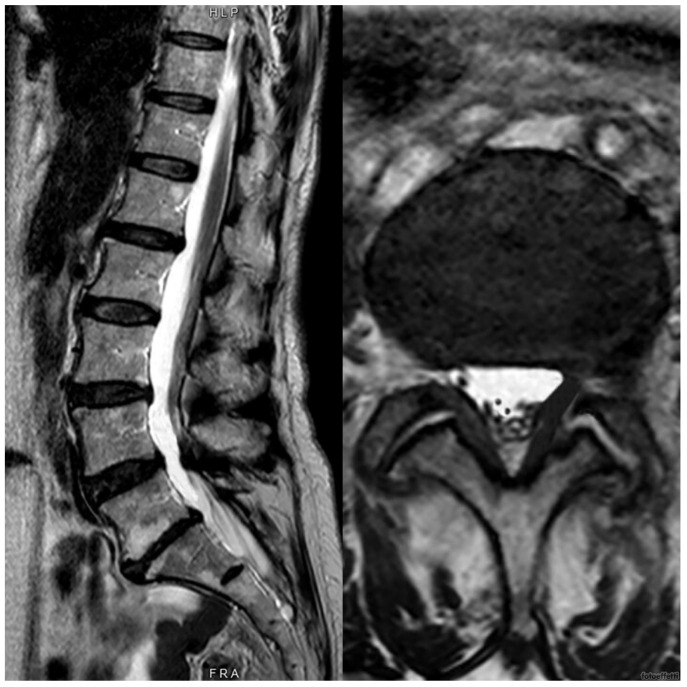
Spontaneous reabsorption of the synovial cyst is described in this MRI of lumbar-sacral spine of the second patient after about 3 months.

In both cases, the synovial cyst was associated with Pfirrmann level 3 degenerative disc disease with a compensatory hypertrophy of lumbar facet joints. The findings and management options were discussed with the patient, who initially opted for surgery. After a clinic review, he was placed on the surgical waiting list. Given that his MRI scans were six months old, a new set of scans was scheduled closer to the operation date. The repeated MRIs conducted about six months later showed a complete resolution of the synovial cysts. As seen in Figure 3 and Figure 4, the synovial cysts previously described had completely disappeared.

Both patients reported a marked improvement in pain symptoms. Only lumbar back pain remained. The surgical procedure was suspended. Patients started a course of physiotherapy with benefit.

## 3. Pathophysiology

A synovial cyst is a cyst containing clear or xanthochromic fluid within a cavity covered with synovium, which communicates with a joint capsule [7]. These cysts are commonly found in the articular regions of the facet joints and, less frequently, within the ligamentum flavum and interspinous ligaments [6]. The underlying pathophysiology of synovial cysts, although still unclear, appears to be related to degenerative changes in the facet joints consistent with repetitive microtrauma [8]. This process leads to the progressive thinning of the joint capsule, eventually causing its wall to fail due to structural weakness. Synovial cysts tend to have a slight female preponderance, with the spine levels most often affected being L4–L5, followed by L5–S1, L3–L4, and L2–L3 [9]. The incidence of lumbar synovial cysts (LSCs) ranges from 0.8% to 2.0% in imaging studies, while it is between 0.01% and 0.8% in patients undergoing lumbar spine surgery [10]. Their pathogenesis is largely debated, although it is generally accepted that they are part of the degenerative spinal process, where instability and spinal injuries play significant roles. The definitive association of these cysts with osteoarthritis (40.5%), spondylolisthesis (43.4%), and disc degeneration (13.2%) highlights their degenerative nature [11].

The most frequent symptom is lower back or radicular pain (55–97%), as observed in our cases, although neurogenic claudication and cauda equina syndrome may also be present. MRI is the gold standard for diagnoses, as it reveals the cystic nature of the lesion and typically shows intermediate signal intensity of extradural masses extending along the medial side of the facet joint, compressing the dural sac [12].

## 4. Management

Clinical management depends primarily on the symptomatology, which very often correlates with the severity of the pathological picture. Radicular pain is the most frequent symptom at clinical presentation. This results in a high percentage of patients who may have weakness of the muscle groups innervated by the root in question [13]. There are numerous treatment strategies for managing lumbar spine synovial cysts. The management of symptomatic LSC should be tailored to each case, depending on the presenting signs and symptoms, radiological findings, the surgeon’s expertise, and the patient’s preferences. Total cyst excision via small flavectomy, as the least invasive approach, should be considered the surgical therapy of choice [14]. The advantage of open surgery is that it allows for extended and appropriate exposure of the cysts and attachment zones, leading to a macroscopic confirmation of excision. However, its disadvantage is the requirement for a medial facetectomy which, when combined with an already degenerated spine, increases the risk of instability and raises the dilemma of whether to use transpedicular screws for attachment.

Minimally invasive spine surgery using tubular retractors for access was described by Foley et al. in 1997 [15]. The paramedian ipsilateral lumbar spine approach minimizes incision size, reduces blood loss, and decreases postoperative pain. Nevertheless, there is still a need for surgical resection of the medial facet, which can accelerate degeneration and increase the possibility of synovial cyst recurrence or segmental instability [16].

The management of symptomatic LSC remains controversial due to the generally unknown natural history of the disease. Surgery is indicated for patients with progressive neurological deficits or intractable pain that does not respond to conservative treatment. However, there is ongoing debate regarding the ideal surgical management approach.

Table 1 contains a case series of the surgical management of symptomatic LSC in the literature; the method of treatment is described as well as the relative outcome. Postoperative outcomes for lumbar spine synovial cysts have been variable with recurrence rates of back pain and leg pain reported at 21.6% and 11.8%, respectively [8]. Conservative treatment has a failure rate of approximately 47% [17]. Careful conservative management typically includes bed rest, analgesia, orthosis, CT-guided percutaneous cyst aspiration, and the injection of epidural and articular facet steroids. Although rare, the spontaneous regression of symptomatic LSC has been documented in a few cases, including our own, demonstrating that spontaneous resorption is a possible outcome and should be considered as part of the cyst’s potential evolution. Factors contributing to spontaneous disappearance may include the extrusion of the cyst’s contents, resorption of the cyst wall, or changes in the local forces that initially led to cyst formation [18]. This case illustrates that the spontaneous resolution of LSC with symptom alleviation is possible, although it remains a rare occurrence [19].

Table 2 details the cases of spontaneous regression of LSC reported in the literature.

Some authors have theorized that spontaneous rupture could be the cause, though the precise mechanism is still uncertain. Others propose that gradual degenerative changes in the facet joint stabilize previously hypermobile facets, leading to a reduction in intra-articular pressure and the subsequent shrinkage of the cyst. In our opinion, the cyst might also undergo degenerative changes, severing its connection with the facet joint and resolving due to a lack of nutrient supply.

As is always the case with oncologic pathology, the timing and type of surgery chosen are central to a surgeon’s work. This is especially true in certain clinical scenarios, such as benign neoplastic brain pathology or oncologic pathology in pediatric patients [38]. The choice of surgical intervention in such settings may involve several variables. A recent systematic review by Benato et al. considered six studies involving a total of 657 patients, comparing outcomes between those treated with lumbar posterior decompression (LPD) and those treated with lumbar decompression and fusion (LDF). It was found that LDF was associated with better outcomes in terms of less postoperative back pain and lower cyst recurrence rates than LPD. No differences were found in terms of complications and reintervention rates, although the follow-up period was notably limited [39]. In the realm of invasive spinal techniques, the tubular approach has recently been validated as both effective and reliable. A study involving 1833 patients compared this approach with the percutaneous approach. It was found that the postoperative length of stay and intraoperative bleeding were lower in the percutaneous groups than in the conventional groups (*p* < 0.05). For patients with lumbar spinal cysts (LSCs) but without clear clinical and imaging evidence of vertebral instability, minimally invasive tubular approaches without fusion may offer the best surgical outcomes [40]. An extremely interesting issue is recurrence, which is reported in our tables as a case of poor outcome. An interesting opportunity to predict it today is represented by artificial intelligence models or clustering methods applied to imaging. [41] Predictive factors for recurrence that are often called out are the facet tilt angle > 45°, canal stenosis > 50%, T2 joint space hyperintensity, and the presence of grade I spondylolisthesis. Recently, work conducted on 89 patients implemented the Lumbar Synovial Cyst Score model, which proved to be a rapid and accurate tool in which the probability of cyst recurrence ranged from <5% for a score of 2 or less to >88% for a score of 7 [42].

## 5. Conclusions

Microsurgical cyst removal is still considered by many researchers to be the most effective treatment option for symptomatic patients. However, reports of the spontaneous regression of synovial cysts have emerged in recent years, suggesting that a purely conservative approach is also plausible. In our opinion, conservative treatment, including analgesics and physical measures, should be attempted before resorting to surgery, provided the neurological deficits are contained and nevertheless not severe. Surgery should therefore become a second-line indication to be undertaken only when conservative treatment pursued reasonably for a few months fails or if neurological deficits develop, requiring rapid management. Further research is needed to better understand the natural history of synovial cysts, improve therapeutic strategies, and establish an appropriate window in which to give a proper indication for surgery.

## Figures and Tables

**Table 1:** Case series addressing surgical management of symptomatic LSC in the literature.

N. of Patients	Mean Age (Years)	M:F Ratio	Outcomes	Reference
15	66, 6	6:9	Excellent 10 Poor 5	Landi et al., 2012 [13]
21	54	14:7	Excellent 11 Good 5 Poor 5	Bashir and Ajani, 2012 [20]
167	63	77:90	117 good 28 poor 22 lost to follow up	Xu et al., 2010 [8]
46	73	17:29	Good 40 Poor 6	Weiner et al., 2007 [21,22]
80	68	41:39	Excellent 36 Poor 32 Recurrence 12	Epstein, 2004 [23]
46	63	8:38	28 Good 10 poor 8 lost to follow up	Pirotte et al., 2003 [24]
29	63	11:19	Excellent 18 Good 6 Lost to Follow Up 5	Banning et al., 2001 [25]
28	60,7	6:22	26 excellent/good 2 fair/poor	Salmon et al., 2001 [26]
19	65	12:7	Excellent 17 Good 2	Trummer et al., 2001 [27]
28	61	NA	Good 24 Recurrence 4	Howington et al., 1999 [4]
8	60	5:3	Excellent 5 Good 3	Jonsson et al., 1999 [28]
56	62,8	24:32	Excellent 40 Poor 16	Sabo et al., 1996 [6]
26	61	12:14	Excellent 15 Good 3 Poor 5	Freidberg et al., 1994 [29]

Excellent: no recurrence at last follow-up, clinic improved by 90%; Good: no recurrence at last follow-up, clinic improved by 50%; Poor: recurrence or delayed fusion for postoperative instability or no clinical improvement or clinical improvement < 50%.

**Table 2:** Cases of spontaneous regression of synovial cysts reported in the literature.

Age/Sex. of Patients	Level	Indications before Reporting Regression	Reference
51 years/female	L4/5	conservative	Nathan J. 2019 [30]
66 years/male	L5/s1	surgery	Priyank Sinhaù 2016 [31]
72 years/male	L4/5	conservative	Pulhorn et al., 2012 [32]
50 years/female	L4/5	surgery	Illerhaus et al. [33]
65 years/male	L4/5	conservative	Ewald et al., 2005 [34]
57 years/ male	L5/s1	surgery	Houten et al., 2003 [18]
58 years/female	L4/5	surgery	Swartz et al., 2003 [35]
15 years/male	L4/5	surgery	Maezawa et al., 2000 [36]
65 years/female	L4/5	surgery	Mercader et al., 1985 [37]

## Data Availability

The data presented in this study are available on request from the corresponding authors.
